# Comparison of Productivity of Colonies of Honey Bees, *Apis mellifera*, Supplemented with Sucrose or High Fructose Corn Syrup

**DOI:** 10.1673/031.013.1901

**Published:** 2013-03-16

**Authors:** Diana Sammataro, Milagra Weiss

**Affiliations:** 1 USDA-ARS Carl Hayden Honey Bee Research Center, 2000 East Allen Road, Tucson, Arizona; 2 Department of Entomology, University of Arizona, Tucson, Arizona

**Keywords:** feeding bees

## Abstract

Honey bee colony feeding trials were conducted to determine whether differential effects of carbohydrate feeding (sucrose syrup (SS) vs. high fructose corn syrup, or HFCS) could be measured between colonies fed exclusively on these syrups. In one experiment, there was a significant difference in mean wax production between the treatment groups and a significant interaction between time and treatment for the colonies confined in a flight arena. On average, the colonies supplied with SS built 7916.7 cm^2^ ± 1015.25 cm^2^ honeycomb, while the colonies supplied with HFCS built 4571.63 cm^2^ ± 786.45 cm^2^. The mean mass of bees supplied with HFCS was 4.65 kg (± 0.97 kg), while those supplied with sucrose had a mean of 8.27 kg (± 1.26). There was no significant difference between treatment groups in terms of brood rearing. Differences in brood production were complicated due to possible nutritional deficiencies experienced by both treatment groups. In the second experiment, colonies supplemented with SS through the winter months at a remote field site exhibited increased spring brood production when compared to colonies fed with HFCS. The differences in adult bee populations were significant, having an overall average of 10.0 ± 1.3 frames of bees fed the sucrose syrup between November 2008 and April 2009, compared to 7.5 ± 1.6 frames of bees fed exclusively on HFCS. For commercial queen beekeepers, feeding the right supplementary carbohydrates could be especially important, given the findings of this study.

## Introduction

Honeybees are facing a myriad of challenges today from interacting stressors, including diseases, parasitic mites, and pesticides, as well as substandard diets, all of which affect their ability to stay healthy (Alaux et al. 2010). Besides these challenges, commercial bee operations (beekeepers who maintain over 1,000 beehives) encounter significant stress from a variety of management practices, including repeated long-distance trucking of colonies to pollinate crops, feeding bees inadequate or insufficient amounts of food, and the questionable ability of modern crop monocultures to provide adequate nutritional diversity for bees. Bees require both proteins and carbohydrates to survive, and proper nutrition is essential to help bees cope with these many pressures. Their protein comes from pollen produced by flowers, which is collected, modified, and stored in the colony as bee bread; it is critical for bee health, development, and survival. Bees also consume large amounts of carbohydrates such as honey, sugar syrup, or flower nectar (Winston 1987), and depend on the products of carbohydrate metabolism to fuel foraging flights (Neukirch 1982; Beenakkers et al. 1984; Kunieda et al. 2006). Carbohydrates also fuel cellular respiration and physical activities such as thermoregulation and locomotion (Chapman 1982).

Floral nectars are the major source of natural carbohydrates for bees, containing among other things, sugars, amino acids, vitamins, organic acids, metal ions, alkaloids, proteins, and oils (Bogdanov et al. 2004; Carter and Thornburg 2004; Park and Thornburg 2009;). The ratio of nectar sugars may depend not only on the anatomy of the plant (Doner 1977) but also on the structures that secrete and conduct sugars (Nicolson and Thornburg 2005). Percival (1965) reported that certain families of plants consistently contained hexose-rich (Brassicaecae and Asteracease) or sucrose-rich (Laminaceae and Rannunculaceae) nectars; sucrose and variable levels of other oligosaccharides are the main sugar components in nectar (Maurizio 1976; Doner 1977; Shuel 1992; De la Barrera and Nobel 2004). Additional sugars found in nectar are non-nutritive because bees are unable to break them down and toxic upon ingestion, especially under laboratory conditions (feeding caged bees). To ensure that a balance of nutrition is obtained, bees require a diversity of plant sources on which to forage.

In order for bees to process and store the carbohydrates (as honey), they first must break down the disaccharides into monosaccharides, because only monosaccharides can pass through the midgut wall into the bee's hemolymph for later use by cells (Crailsheim 1988). Therefore, all the complex sugars bees ingest must be enzymatically transformed in order to become bioavailable to them (Hausmann et al. 2005). Results from sequencing the genome of the honey bee (Honey Bee Genome Sequencing Consortium 2006) have identified genes that encode carbohydrate-metabolizing enzymes. Other researchers have identified proteins from the food and salivary glands related to the metabolism of carbohydrates and energy production (Santos et al. 2005; Fujita et al. 2010). For example, the enzyme invertase converts sucrose into glucose and fructose (White et al. 1963; Simpson et al. 1968; Winston 1987; Kubo et al. 1996; Ohashi et al. 1996, 1997, 1999; Kunieda et al. 2006). Bees not only convert the sugars in the nectar, but add microorganisms and reduce the water content to prevent fermentation. The final product, honey, is stable and normally contains the following proportions: fructose (38.2%), glucose (31.3%), sucrose (1.3%), maltose (7.1%), water (17.2%), other components (3.1%) (White 1980).

### High Fructose Corn Syrup vs. Sucrose

Commercial bee colonies are moved into many different locations for pollination, and some areas may not have sufficient forage. In this situation, bees depend on the beekeeper to provide them with food. Because large numbers of colonies (sometimes over 20,000) can be temporarily held in a single location, there is a growing reliance on mass-feeding bees carbohydrates and proteins. Though honey has long been considered to be the “ideal bee feed” (Bailey 1966), researchers and beekeepers have recognized that sucrose may be a better sugar supplement (Herbert 1992). In some situations, especially in noncommercial operations, providing additional frames of sealed honey to a carbohydratedeficient colony might be the least laborintensive method, but this practice can also increase the risk of spreading American foulbrood disease, a spore-forming bacterial disease commonly found in honey. By feeding sugar syrup, spreading this disease is avoided (Goodwin 1997; Sammataro and Avitabile 2011). However, in commercial operations, there are certain disadvantages to feeding large quantities of sucrose syrup (SS), such as making the syrup, which requires a significant labor input (Goodwin 1997), and the tendency of sucrose to crystallize and ferment, making long-term storage difficult. During dearth time, such as winter, feeding serves as complete substitutes for natural forage, and high fructose corn syrup (HFCS) is commonly used.

HFCS is produced by multi-step enzyme hydrolysis of corn starch into a glucose/fructose mixture (Long 1986; Hanover and White 1993) that was developed in the late 1960s (Schorin 2005). The original method used acids to convert the corn starch; the acids commonly used were sodium hydroxide (NaOH) and hydrochloric acid (HCl) (LeBlanc et al. 2009). Newer processes include using chemicals and genetically modified bacteria, such as α-amylase, glucoamylase, and others (LeBlanc et al., 2009). HFCS is processed into three common formulations called HFCS-90, HFCS-55, and HFCS-42, which are named for the percentage of the sugar fraction that is fructose (Long 1986). The remainder of the sugar fraction is glucose and some traces of unnamed oligosaccharides. These water-based formulations range from 71–77% dissolved solids. With the enzyme-processing of corn starch optimized (Linko et al. 1977), HFCS has become a widely-available and cheap carbohydrate source (Hanover and White 1993) and a common sweetening agent for human consumption. Currently, HFCS is being scrutinized because of reports that human diets high in HFCS are related to serious health issues, such as the development of obesity, diabetes, and hypertension (Bocarsly et al. 2010; Ferder et al. 2010; Nseir et al. 2010). As a result of negative press, the industry is changing its name from High Fructose Corn Syrup to just Corn Sugar (Nestle 2010; Parker-Post 2010).

HFCS became available as a cheap source of bee feed starting in the 1970s (Barker and Lehner 1974, 1978). Due to the ease of handling HFCS over mixing sucrose solutions and often cheaper pricing, the use of HFCS for bee feed increased (Herbert 1992). Because the sugar profile of HFCS is very similar to that of honey (Bogdanov et al. 2008) and the use of HFCS in apiculture is so widespread, it is unfortunately sometimes used as a honey adulterant, so much so that there has been a significant amount of work for developing techniques to detect HFCS in honey (Abdel-Aal et al. 1993; Megherbi et al. 2009). Questions about the safety HFCS as bee food were raised soon after it became available, because beekeepers reported mixed results from feeding it (Bailey 1966; Johansson and Johansson 1976; Anon 1996; Sanford 1997). In addition, researchers found decreased longevity in worker bees maintained in the laboratory on HFCS as compared to honey (Barker and Lehner 1978) or to SS (Weiss 2009). More recently, the news of alarming bee mortality called Colony Collapse Disorder or CCD (vanEngelsdorp et al. 2007, 2010) suggested there might be deleterious effects of HFCS feeding. Reports of bees not feeding or dying after being fed were mentioned by some beekeepers (Oliver 2007). HFCS can cause other problems, such as the formation of toxins as a result of heat, the chemical properties of fructose, and the low pH of HFCS (Kim et al. 1995; LeBlanc et al. 2009). These conditions can promote the hydration product and known bee toxin, hydroxymethylfurfural (HMF), which readily forms during high heat storage conditions. Samples of bee feed from various commercial beekeeping operations, where the syrup was stored in outside tanks, have confirmed this hypothesis (Weiss 2009; Ruiz-Matute et al. 2010). Non-commercial beekeepers rarely use HFCS and therefore are not usually affected.

As a result of these mixed results, new investigations of HFCS as a bee feed were needed. The purpose of this research was to determine if feeding HFCS was detrimental to bees in the long term. Colony-level trials were performed in order to establish whether earlier work on the decreased longevity of individual worker bees fed on HFCS in caged-bee experiments (Weiss 2009) would manifest as a decrease in colony productivity, measured in differences in adult bee and brood populations. Studies using caged bees are limited and may reveal health effects caused by stresses that bees would not normally experience in the hive. Testing this syrup in field experiments would yield more useful information. In the first experiment, colonies were fed exclusively on HFCS or SS in a controlled environment (flight arena); in the second trial, overwintering colonies were fed in order to determine which source of carbohydrate was best for bees.

## Materials and Methods

### Experiment One: Colony-founding

Swarm colonies were produced after the protocol by Mattila and Seeley (2007). Ten colonies were established with mated queens from a commercial queen breeder (Palo Cedro, CA) at the Carl Hayden Bee Research Center, Tucson, AZ, apiary. The queen viability (egg laying) was monitored for 4 week. During this period, adjustments were made to equalize colony strength by redistributing frames of stored honey and brood. The bees were treated for mites using acaricide strips (Apistan, file://localhost/htt/::medivetpharmaceuticals.webs.com:apistan.htm). On June 19, 2008, all queens were individually caged within their colonies, and in the following pre-dawn hours, each colony was shaken from its frames, weighed, and transferred to a screened swarm box with its queen. The swarm box used was an empty nucleus (nuc) hive body with a mesh bottom, used to collect swarms. These shaken bees were held for 3 days in a dark room and supplied with a 50% sucrose solution, after which they were transferred onto 5 frames of undrawn plastic foundation (Rite Cell, Mann Lake Ltd., http://www.mannlakeltd.com/) and placed in new nucleus hive equipment.

After 3 days, 10 of the 5-frame nucs went into an enclosed flight arena (modified Quonset style greenhouse) at the University of Arizona Agricultural Research Center, adjacent to the Carl Hayden Bee Research Center. The 10 nucs in the arena were randomly divided into two groups of 5 for feeding treatments. All colonies were fitted with top feeders and randomly assigned a treatment of either commercial bee feed HFCS or SS. Corn Sweet HFCS55 was used as the HFCS feed (no HMF formation was found), and sucrose mixed first with hot tap water (hot enough to dissolve the sugar crystals) then cooled, was the SS feed. The syrup solids were then equalized (both solutions, 50% solids v/v) and nucs were continually supplied on a weekly basis. MegaBee® (Castle Dome Solutions, http://www.castledomesolutions.com/) patties, which contain no natural pollen, were fed *ad libitum* as a protein supplement. Thereafter, the brood area, new wax areas, and adult bee populations were measured, using a 1 inchsquare metal grid, every 12 days to ensure measurement of a unique brood patch. Colony mass (including bee biomass, wax, and food storage) was monitored every 12 days using a portable platform postal scale (Acculab, Bradford, MA). Frames of bees, honey, and pollen were recorded until mid-August. Measurements were analyzed using repeated measures ANOVA; where Mauchly's test of sphericity was significant, the Greenhouse-Geisser correction to the degrees of freedom was used.

### Experiment Two: Overwintering

Ten deep Langstroth colonies of approximately equal strength and headed by sister queens of the same generation were moved from the Carl Hayden Bee Research Center apiary to the Desert Grasslands Station in the Santa Rita Experimental Range (Green Valley, AZ) in November 2008. The goal was to determine the change between the two syrup treatments over the 6 months by measuring brood, bee populations, pollen, and honey frames (indications of colony strength). The average temperature during this period (November through April) was 14.5° C (range 11.1° to 18.3° C), with a total precipitation of 92.964 mm. Weather data were obtained from the University of Arizona (http://ag.arizona.edu/SRER/index.html) and the USDA Southwest Watershed Resource Center, Tucson, AZ. Few flowers bloom during this time, so there is limited forage until April; bee populations generally do not expand until after May.

The colonies were standardized with 3 frames each of stored honey and 3 frames of empty drawn comb; the rest of the frames were undrawn plastic (RiteCell). The colonies were each fitted with a hive-top feeder and provided a randomly assigned treatment of either HFCS or SS for 1 month. In an effort to compare HFCS and SS as carbohydrate sources for winter and spring, colonies were heavily supplemented with carbohydrates and patties *ad libitum* in November. The syrup and patties were consumed between the weekly spring feeding. Pollen patties were made of local bee-collected pollen mixed with syrup to form the patty. Pollen was used because natural pollen could also be collected by bees and pollen was more acceptable than the MegaBee patties. In December, all feeding stopped and the hive-top feeders were removed. In February, the colonies were again fitted with hive-top feeders, provided with pollen patties, and fed the same syrup treatments (in equal volumes) as previously. Spring adult population, brood, and food storage were recorded as before. Capped brood areas were measured seven times using a 1-inch square grid; measurements were made once at the time of initial colony placement and thereafter approximately twice per month, with at least 12 days between measurement events to ensure that unique patches of capped brood were counted each time. Frames of bees, honey, and bee bread were also recorded. Data were analyzed as in the colony-founding experiment.

## Results

### Experiment One: Colony-founding

Within two weeks of colony installation inside the foraging arena, two of the colonies assigned to the sucrose treatment failed (bees absconding) and were removed from the study. Three colonies assigned SS and 5 colonies assigned HFCS remained in the study, and all data for the colony-founding experiment refers to these bees maintained in the arena. There was a significant difference in mean wax production between the treatment groups (F_1,6_ = 6.850, *p* = 0.040) and a significant interaction between time and treatment (F_1,6_ = 6.266, *p* = 0.042) (Table 1). On average, the colonies supplied with HFCS built 4571.63 cm^2^ ± 786.45 cm^2^ of honeycomb while the colonies supplied with SS built 7916.74cm^2^ ± 1015.25 cm^2^ (Table 1). The difference in food storage (syrup stored in the comb), measured as colony mass, was highly suggestive though not significantly different (F_1,6_ = 5.476, *p* = 0.058). The mean mass of colonies (total weight) supplied with HFCS was 4.65 kg (± 0.95 kg), while those supplied with SS had a mean of 8.27 kg (± 1.26). There was no significant difference between treatment groups in terms of brood rearing (F_1,6_ = 1.250, *p* = 0.306). After 6 brood cycles (126 days) within the foraging arena, general failure was recognized in all colonies and the study was terminated. This failure included an almost complete lack of brood rearing and severely diminished adult populations despite prolific egg-laying by the queens; samples were sent for disease identification, but all came back negative.

### Experiment Two: Overwintering

Brood rearing was different between colonies that over-wintered on HFCS and colonies that over-wintered on SS (F1,8 = 5.693, *p* = 0.044). The interaction between time (sample period) and winter feed treatment was not significant (F_1,8_ = 1.442, *p* = 0.27). The mean amount of brood produced in the spring by colonies feeding on HFCS was 1889.03 cm^2^ ± 467.2 cm^2^, while mean brood production for colonies feeding on SS was 3045.16cm^2^ ± 528.7 cm^2^. There was no significant difference between treatments in terms of frames of pollen (F_1,8_ = 1.237, *p* = 0.298) or honey (F_1,8_ = 0.017, *p* = 0.899). However, the adult bee populations differed (F_1,8_ = 5.011, *p* = 0.056), with an overall average of 10.0 ± 1.3 frames of bees fed SS between November 2008 and April 2009, compared to 7.5 ± 0.16 frames of bees fed HFCS.

## Discussion

SS has long been recognized by beekeepers as having a stimulatory effect, such as an increase in egg-laying and pollen-gathering activities (Barker 1971), as well as increased hygienic behavior (M. Spivak, Department of Entomology, University of Minnesota, personal communication). Free and Spencer-Booth (1961) observed bees switching their foraging strategy after sucrose feeding; they noted a decrease in the number of nectar foraging bees and an increase in pollen foraging bees to support colony brood rearing. However, HFCS has become more widely used to feed colonies because of it lower expense and ease of handling; it is usually delivered by tanker trucks already mixed and ready to feed. The most common form of the syrup purchased by beekeepers is HFCS-55, which is popular because crystallization is avoided and the level of dissolved solids, as well as low pH and high osmotic pressure, resists fermentation and bacterial contamination (Schorin 2005). Supplemental feeding is especially important when bees are moved into orchards before there is sufficient bloom or during inclement weather. Extra feeding is needed at other times, such as during package bee installation on undrawn comb, to supplement winter stores, to stimulate early spring brood rearing, to encourage colony expansion for future colony divisions, and during queen rearing and production operations (Herbert 1992).

In the findings of the present study, HFCS and SS both supported colony establishment and neither caused an acute toxic effect in the field, although SS appeared to have more stimulatory effects on colony establishment activities. From previous work, it was found that HFCS syrup, and the honey that bees store when fed it, contains other oligosaccharides such as fructosyl-fructoses and fructosyl-glucoses, as well as other components (Dufault et al. 2009; Ruiz-Matatue 2010). These additional components may contribute to the high mortality in caged bees (Weiss 2009), as well as the lower attraction to bees in the field studies. These ingredients may be toxic or they could be more difficult for bees to metabolize and digest, and could thus interfere with their ability to produce wax or brood food. Further testing is needed.

Current information on the effect of HFCS on honey bees, however, is limited. Early research reported that bees ate the syrup with no problems (Barker and Lehner 1978; Rinderer and Baxter 1980), and others found that it was toxic to bees (Bailey 1966; Jachimowicz and El Sherbiny 1975; Johansson and Johansson 1977). Barker and Lehner (1978) fed bees various sugars, and found that bees survived better, produced the same amount of wax per bee, and capped more honeycomb cells on sucrose than HFCS, honey, or grape syrup (which is high in fructose). They also found that bees lived longer when supplied with SS. From the caged bee experiments in the present study, it was found that day-old bees lived significantly longer on SS than those fed on HFCS. In addition, there was a tendency for the groups provided with SS to maintain a slightly higher head protein level at day 4 than those maintained on various dilutions of HFCS (Weiss 2009). In the study in the flight arena, it was observed that after approximately six weeks, the number of capped brood cells diminished in all colonies, in spite of prolific egg-laying by all queens. Bees that are denied access to the outdoor environment do not thrive, and it was hypothesized that the protein source could have been insufficient, or that the beneficial microbes needed for the bees to process food were missing in the restricted arena (or a combination of both). Other factors could also be responsible.

The field tests done by Severson and Erikson (1984), who fed HFCS as a supplementary feed for colonies overwintered in Wisconsin, found a slight increase in spring brood production from colonies provided with SS as compared to HFCS-42 and HFCS-55. The colonies did not significantly differ in spring cluster size, worker dry weights, or seasonlong honey production. They concluded that overall, HFCS did not hinder productivity of the colonies over the long term and was an acceptable supplementary feed. However, no other field test information is available. The survival of the colonies in the present study in the overwintering experiment was not greatly affected by either of the feeds, and the spring adult populations and food storage did not significantly differ. However, brood production was significantly greater for colonies provided with SS. Today, commercial beekeepers use supplemental syrup not only to prevent starvation, but also to encourage colony expansion in times of dearth or for spring build-up. Increased brood rearing means an increase in pollination efficiency, since pollen foragers are more efficient pollinators than nectar foragers (Goodwin 1997) and pollen is essential for rearing brood. It also means that the beekeepers will be paid more for colonies that are more populous (Mussen 2010).

While HFCS is an acceptable bee feed, a cautionary note should be mentioned here. HFCS was sampled from several commercial beekeepers that stored their syrup in outdoor tanks, and these samples were found to contain high amounts of HMF (Weiss 2009; Ruiz-Matute et al. 2010). When HFCS is stored for a long time, especially where the syrup can be easily overheated, HMF will form (LeBlanc et al. 2009). The presence of HMF is known to cause dark coloring to honey, and is toxic to honey bees (Kim et al. 1995; LeBlanc et al. 2009). If HFCS is going to be used by commercial bee operations, it must be stored in a temperature-controlled facility and not mixed with old, unused syrup or with water. Currently, many commercial operations are now mixing HFCS with sucrose to prevent the formation of HMF and mitigate the effects of HFCS alone.

The second observation that was made is that bees raised more brood on, and were more attracted to, SS. This is an important factor for commercial beekeepers that are feeding bees in the later winter months and want large bee populations in time for the spring pollination season. Today, thousands of colonies are fed HFCS, especially in staging areas before almond bloom in California, when no other natural forage is available. Beekeepers feed their colonies to build up bee populations in the few months before bees are moved into the orchards for pollination. When bees are leased for their pollination services, the stronger, more populous colonies are worth more money. For example, a colony of bees with at least six frames of bees and brood can collect 1.5× more pollen than a four-frame colony; an eight-framer collects 2× more (Mussen 2010). Almond growers want to rent colonies having either an eight-frame average or, in some cases, an eight-frame minimum bee population. An eight-framer is worth an average of $144 each (Traynor 1980; Mussen 2010). Growers require one to two colonies per acre (0.4046 hectares) of orchard. If HFCS is used as the sole carbohydrate source during these times, the decrease in brood production could translate into a proportionately lower bee population overall. So, for commercial operations, while HFCS did no harm, beekeepers feeding SS on a large scale may have a significantly higher overall return in larger bee populations.

**Table 1.**
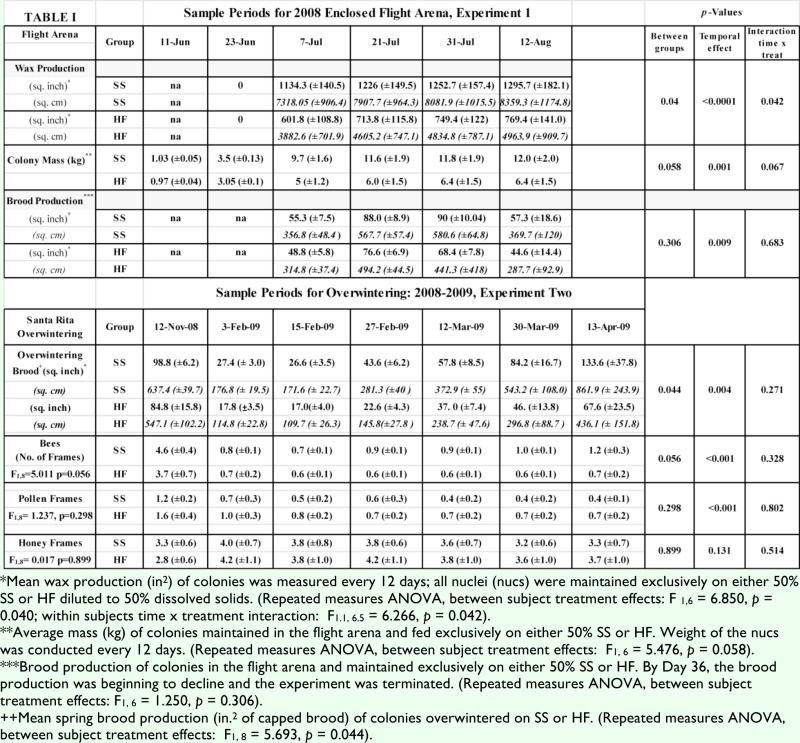
Wax production, colony mass, and brood production in the Flight Arena and in the overwintering colonies at Santa Rita. SS = sucrose syrup, HF = high fructose corn syrup.

##

**Abbreviations**
HFCS: high fructose corn syrup
HMF: hydroxymethylfurfural
Nuc: nucleus
SS: sucrose syrup
